# Optimising biocatalyst design for obtaining high transesterification activity by α-chymotrypsin in non-aqueous media

**DOI:** 10.1186/1752-153X-2-2

**Published:** 2008-02-12

**Authors:** Kusum Solanki, Munishwar Nath Gupta

**Affiliations:** 1 Department of Chemistry, Indian Institute of Technology Delhi, Hauz Khas, New Delhi, 110016, India

## Abstract

**Background:**

Enzymes are often used in organic solvents for catalyzing organic synthesis. Two enzyme preparations, EPRP (enzyme precipitated and rinsed with n-propanol) and PCMC (protein coated microcrystals) show much higher activities than lyophilized powders in such systems. Both preparations involve precipitation by an organic solvent. The clear understanding of why these preparations show higher catalytic activity than lyophilized powders in organic solvents is not available.

**Results:**

It was found that EPRPs of α-chymotrypsin prepared by precipitation with *n*-propanol in the presence of trehalose contained substantial amount of trehalose (even though trehalose alone at these lower concentrations was not precipitated by *n*-propanol). The presence of trehalose in these EPRPs resulted in much higher transesterification rates (45.2 nmoles mg^-1^min^-1^) as compared with EPRPs prepared in the absence of trehalose (16.6 nmoles mg^-1^min^-1^) in octane. Both kinds of EPRPs gave similar initial transesterification rates in acetonitrile. Use of higher concentrations of trehalose (when trehalose alone also precipitates out), resulted in the formation of PCMCs, which showed higher transesterification rates in both octane and acetonitrile. SEM analysis showed the relative sizes of various preparations. Presence of trehalose resulted in EPRPs of smaller sizes.

**Conclusion:**

The two different forms of enzymes (EPRP and PCMC) known to show higher activity in organic solvents were found to be different only in the way the low molecular weight additive was present along with the protein. Therefore, the enhancement in the transesterification activity in EPRPs prepared in the presence of trehalose was due to: (a) better retention of essential water layer for catalysis due to the presence of the sugar. This effect disappeared where the reaction media was polar as the polar solvent (acetonitrile) is more effective in stripping off the water from the enzyme; (b) reduction in particle size as revealed by SEM. In the case of PCMC, the enhancement in the initial rates was due to an increase in the surface area of the biocatalyst since protein is coated over the core material (trehalose or salt).

It is hoped that the insight gained in this work would help in a better understanding for designing high activity biocatalyst preparation of non-aqueous media.

## Background

The possibility of using enzymes in low water containing organic solvents has expanded their applications in organic synthesis [1-4]. In many cases, improved regioselectivity and enantioselectivity have been observed [5,6]. The considerable success obtained in such applications in numerous cases has obscured the fact that reaction rates in such media tend to be extremely slow. For an organic chemist, reaction times of 24–72 h are not a novelty as many chemical transformations, catalysed by chemical catalysts, often take that kind of time. Hence, the early successes with use of enzymes in organic synthesis in non-aqueous media had created considerable excitement [7,8]. In the last decade or so, it has been realized that reaction rates in non-aqueous media are considerably lower than those in the conventional aqueous media. For example, the catalytic efficiency of subtilisin for hydrolytic reactions (esterase activity in water) is 5.94 × 10^3 ^M^-1^s^-1 ^whereas its catalytic efficiency for transesterification using the same ester (in hexane) is merely 1.04 × 10^-1 ^M^-1^s^-1^[9]. In fact, formation of alkyl esters of long chain fatty acids (biodiesel) takes only an hour or so when alkali is used as the catalyst; lipase catalyzed formation takes more than 24 h [10]. Hence the common perception (based upon all our experience in conventional enzymology) that enzymes are more efficient catalysts than chemical catalysts is often not valid in non-aqueous enzymology. Yet, there are many advantages of using enzymes as catalysts. Hence, the reasons for slowness of enzyme catalyzed reactions in organic solvents have been investigated [9]. Among the many reasons, an important one to emerge is that in lyophilized powders (the frequently used forms of enzymes employed in organic solvents), enzymes, in fact, have conformations which are very different from native structures. While these conformational changes are reversed when enzymes are dissolved in water, these remain permanant when enzymes are used as powders in organic solvents. Again, numerous strategies have been described in the literature to obtain enzyme activation in non-aqueous media [9]. Two simple and elegant strategies are based upon avoiding lyophilization. In propanol rinsed enzyme preparations (PREP) [11], the bulk water from immobilized enzyme is removed by rinsing with n-propanol. In protein coated microcrystals (PCMC) [12], protein is coprecipitated along with a salt (or another low molecular weight organic compound) by an organic solvent. Both PREP and PCMCs show much higher rates as compared to lyophilized powders in organic solvents [13,14]

Few years back, we described EPRPs [15]. The idea was to merely adapt PREP concept to free enzymes. It was found that more active EPRPs were formed if an optimum amount of buffer salts were present [15]. The role of the buffer salts in that preliminary work was not investigated. One possibility was that this optimum amount of salt prevented any structural damage to the protein structure. Many small molecular weight additives are known to protect enzymes during exposure to high temperature [16], during lyophilization [17] and during catalysis in organic solvents [18,19]. Disaccharides have been found to be an effective additive as a protector of enzymes activity during both thermal stress [20] as well as lyophilization [9,21]. The present work was initiated to find out whether presence of disaccharides offers any protection to the enzyme during its exposure to organic solvent during precipitation by an organic solvent for formation of EPRPs.

## Results and discussion

α-chymotrypsin has been used often as a model enzyme to understand enzyme function in low water containing media [15,18,22]. Triantafyllou et. al. [18], briefly mention that lyophilized powders of α-chymotrypsin when washed with pyridine retained more transesterification activity (in organic medium) when sorbitol was present. As this result was reported merely as a control in their studies, quite understandably no clear explanation of this observation was provided. Table 1 shows that the presence of various disaccharides during precipitation of α-chymotrypsin by n-propanol for formation of EPRP [15] led to the enzyme preparations which showed higher transesterification activity in anhydrous octane as compared to EPRPs in the absence of disaccharides. Trehalose was found to be best additive. The above experiments were carried out with 1 percent (wv^-1^) trehalose. Addition of n-propanol to the trehalose (1 percent wv^-1^) solution did not precipitate trehalose. Curiously enough, the presence of PEG (a known cryoprotectant during lyophilization) [21] alone or in combination with trehalose did not lead to significant improvement in the transesterification activity of resultant EPRP. Also, FT-IR (in amide I region) of the suspensions of the precipitate obtained with or without the presence of 5 percent trehalose were identical (data not shown). FT-IR has been used effectively for showing the role of lyoprotectants and cryoprotectants during lyophilization [17]. This prompted us to further investigate the effect of trehalose.

**Table 1:** The effects of 1 percent (wv^-1^) disaccharides and PEG-6000 on the activity (initial rates) of α-chymotrypsin precipitated by *n*-propanol.

**Disaccharide**	**Initial rate **(nmoles mg^-1^min^-1^.)
None	16.6
Trehalose	33.5
Trehalose + PEG-6000(1 percent wv^-1^)	38.1
Sucrose	26.0
Lactose	33.7
PEG-6000 (1 percent wv^-1^)	19.6

Initial rates were calculated from aliquots taken over 10 to 60 min. The percentage conversions during these time periods were 1–30 percent and in a linear range.

Table 2 shows the results with use of increasing amounts of trehalose during EPRP preparation. At 15 percent (wv^-1^) of trehalose, the disaccharide (even when present alone without any protein) started precipitating out as a crystalline material. At this point the preparative method became identical to the one described for preparation of protein coated microcrystals (PCMCs) [12]. PCMCs using sugars as crystallization matrix have been described [12]. Thus, these results in a way describe 'evolution' of PCMCs from EPRPs. Interestingly enough, there was an optimum concentration of trehalose around 30–40 percent (wv^-1^) beyond which transesterification activity of PCMC suddenly dropped. PCMCs with both trehalose and many other low molecular weight organic compounds such as potassium sulphate have been described. For comparison, the transesterification activity of PCMCs made with potassium sulphate is also given. The PCMCs prepared from trehalose gave marginally better activity.

**Table 2:** The effects of trehalose concentration on the activity (initial rates) of the α-chymotrypsin precipitated by *n*-propanol in octane. The experiments were carried out in triplicate. The percentage error in a set was within 7 percent.

Starting trehalose (percent wv^-1^)	percent trehalose precipitated	Enzyme loading^a ^(weight percentage)	Initial rate^b ^(nmoles mg^-1^min^-1^)	Visual morphology of enzyme precipitate	Precipitation of trehalose in propanol (control)
0	0	-	16.6	Powder (EPRP)	No precipitation
2	65	38.9	40.0	Powder (EPRP)	No precipitation
5	90	15.5	45.2	Powder (EPRP)	No precipitation
10	94	7.5	63.2	Powder-crystalline	No precipitation
15	88	5.9	120.0	Crystalline	Precipitation
20	85	4.6	125.1	Crystalline	Precipitation
25	78	4.0	143.2	Crystalline	Precipitation
30	76	3.5	150.5	Crystalline	Precipitation
35	72	3.1	152.0	Crystalline	Precipitation
40	70	2.8	151.2	Crystalline	Precipitation
45	65	2.3	96.4	Crystalline	Precipitation
Saturated trehalose solution	60	1.5	87.4	Crystalline	Precipitation
PCMC (K_2_SO_4_)	-	5.8	132.5	Crystalline	Precipitation

^a ^$\text{percent enzyme (w/w)}=\frac{\text{mg protein precipitated}}{\text{(mg of protein precipitated }+\text{ mg of core material precipitated)}}\times 100$

The amount of protein precipitated in each case was 0.83 mg.

^b^Initial rates were calculated from the aliquots taken over 10 to 60 min. The percentage conversions during these time periods were 1–30 percent and in a linear range.

**Figure 1:**
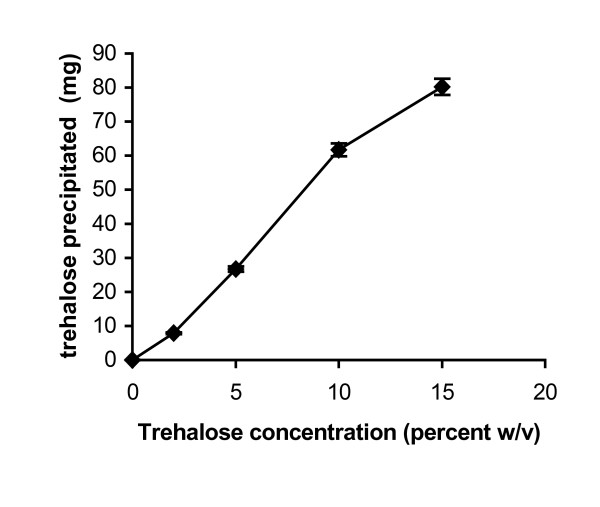
**Precipitation of trehalose in the presence of protein**. A solution of α-chymotrypsin (1 mg in 100 μl of 10 mM potassium phosphate buffer, pH 7.8) containing different concentrations of trehalose, was precipitated by 3 ml of n-propanol at 4°C with constant shaking at 150 rpm. The amount of precipitated trehalose was determined by HPLC as described in the experimental section. The experiments were carried out in triplicate. The error bar represents the variation in the readings.

An analysis of the enzyme precipitation obtained in the presence of 0–10 percent (wv^-1^) trehalose was carried out on HPLC gel filteration column (Fig. 1). It showed that when enzyme was present, trehalose precipitated along with it. The actual presence of trehalose during the transesterification (in octane) meant that the enhancement in transesterification activity was at least partly due to its role during the catalysis. The earlier work with sugars like sorbitol indicates that water uptake by the enzyme preparation increases when sorbitol is present during the exposure to a fixed a_w _[19]. It has been also mentioned that such additives actually work as an immobilization matrix and disperse the enzyme powders (and lead to less mass transfer constraints during the reaction) [18,23]. All these results/observations pertain to lyophilized powders.

In all cases, 0.83 mg of enzyme was precipitated. Initially, until the addition of 10 percent (w/v) trehalose, the percentage amount of trehalose precipitated increased with the increase in the starting concentration of trehalose. As trehalose alone did not precipitate until this concentration, it can be assumed that the presence of the protein caused the trehalose to co-precipitate. Beyond this percentage (w/v) trehalose, trehalose precipitates as micro crystalline material even when present alone. This precipitation is much faster than protein precipitation which leads to a protein being coated on the microcrystals [12]. It is interesting to note that over this concentration range, percentage trehalose getting precipitated decreased with the increase in the percentage (w/v) of starting trehalose. Enzyme loading continued to decrease progressively throughout, as the actual amount of trehalose being precipitated continued to progressively increase.

In the present context, the presence of trehalose in EPRPs presumably helped the enzyme in retaining the larger amount of water during the reaction. In order to check that; EPRPs prepared in the presence and absence of trehalose were used for transesterification in acetonitrile (Table 3). It is well established that enzymes in nearly anhydrous organic solvents require a minimum amount of water to retain conformational flexibility. It is also agreed that polar solvents like acetonitrile strip off water from the enzyme preparations and this is largely responsible for lower reaction rates in more polar media [1-4]. It was seen that the presence of trehalose did not affect the initial rates obtained with EPRPs in acetonitrile. The presence of trehalose could not help enzyme preparation in retaining the bound water required for good catalytic performance in the more polar medium.

**Table 3:** The effects of trehalose concentration on the activity (initial rates) of α-chymotrypsin precipitated by *n*-propanol in acetonitrile.

Trehalose concentration (wv^-1^)	Initial rate (nmoles mg^-1^min^-1^)
0	0.12
10	0.16
25	0.15
Saturated trehalose	1.15
Saturated K_2_S0_4_	0.96

Initial rates were calculated from the aliquots taken over 0.5 to 3 h. The percentage conversions during these time periods were 1–10 percent and in a linear range.

**Figure 2:**
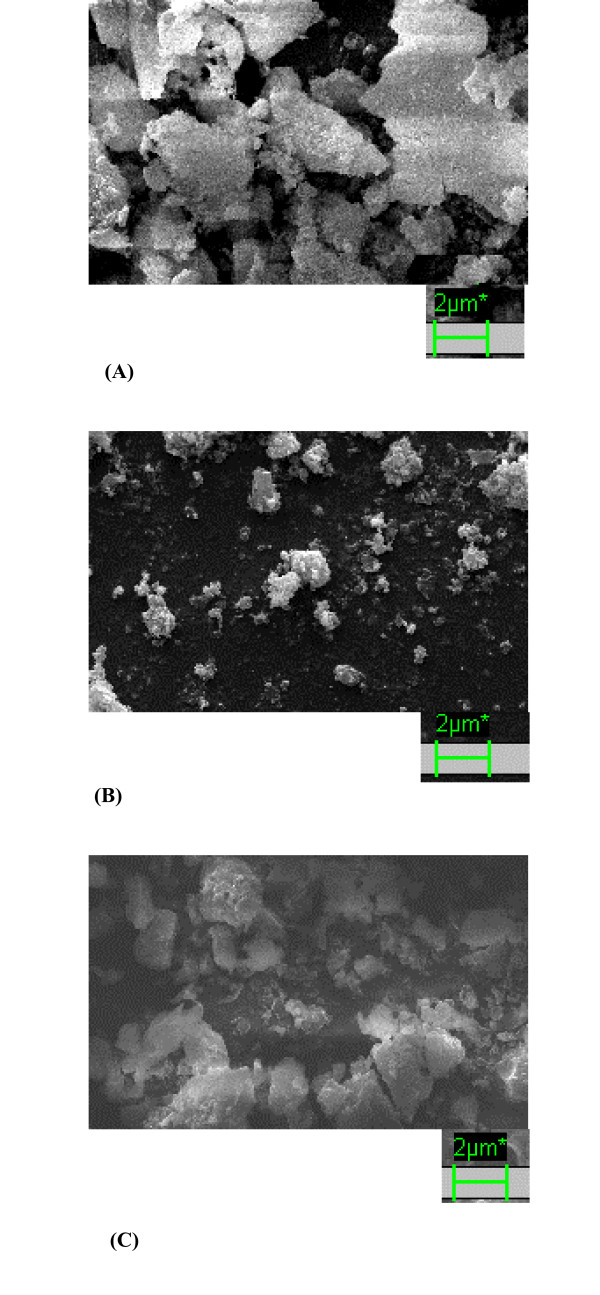
**Scanning electron microscopy (SEM) images of (A) EPRP of α-chymotrypsin (0 percent trehalose) precipitated into *n*-propanol (magnification: 5000 ×); (B) EPRP of α-chymotrypsin (5 percent trehalose) precipitated into *n*-propanol (magnification: 5000 ×); (C) PCMC of α-chymotrypsin (40 percent trehalose) precipitated into *n*-propanol (magnification: 5000 ×)**. SEM was carried out on a Zeiss EVO50 scanning electron microscope. Samples were dried by rinsing with anhydrous propanol, placed on a sample holder, and coated with silver before being scanned *in vacuo*.

The results given in Table 2 and Table 3 also clearly establishes the superiority of PCMCs over EPRPs. PCMCs were reported to give higher activity than lyophilized powders as in PCMCs, "the organic solvent dehydrates the enzymes by mechanism that minimizes denaturation and appears to leave the majority of enzyme molecules in active conformation". That mechanism as per results obtained in the present work should apply equally well to EPRPs. However, EPRPs show much lower activity than PCMCs. The present work, by establishing close relationship between EPRPs and PCMCs, also indicates that additional mechanisms for higher activity established by PCMC should exist. Presumably, the enzyme spread as a coat over the inner microcrystal core offers larger catalytic surface area. This also explains why potassium sulphate works just as well as trehalose as the core matrix in PCMCs. Fig. 2 shows the SEMs of EPRP prepared without the presence of trehalose (Fig. 2A), EPRP prepared with presence of 5 percent trehalose (Fig. 2B) and PCMC (Fig. 2C). The range of particle sizes of EPRP prepared in the presence of trehalose was found to be substantially smaller. This raises the possibility that higher surface areas available with biocatalysts of smaller sizes also contributed to higher transesterification activity. PCMCs (Fig. 2C), of course looked crystalline in nature. PCMCs, though, of bigger size range have enzyme coated over the surface. Thus, the enhancement of biocatalyst surface is much larger than even EPRPs of smaller size range.

## Conclusion

The two different forms of enzymes (EPRP and PCMC) known to show higher activity in organic solvents were found to be different only in the way the low molecular weight additive was present along with the protein. In PCMC, a more defined microcrystals compound is formed. This, of course, is well described in a number of earlier papers. [12,15].

Based upon FT-IR data, it appears that the presence of trehalose did not affect the structure of the enzyme after precipitation. So, the enhancement in the transesterification activity in EPRPs prepared in the presence of trehalose was due to the actual presence of trehalose during the reaction. This was largely due to (a) better retention of essential water layer for catalysis due to the presence of the sugar [19]. This effect disappeared where reaction media was polar as the polar solvent (acetonitrile) is more effective in stripping off the water from the enzyme [1-4], (b) reduction in particle size revealed by SEM.

As trehalose concentration became high enough for trehalose to precipitate out even when present alone, trehalose precipitated faster than the protein. This led to the well known preparation called PCMCs. As both EPRPs and PCMCs described here contained trehalose, the observed higher initial rates with PCMCs can be attributed to the fact that in these biocatalyst designs, the protein was coated over the crystalline material. This created a large biocatalyst surface. It also agrees with the observation that both trehalose and potassium sulphate worked more or less the same as the core material in PCMCs.

It is hoped that the insight gained in this work would help in a better understanding for designing high activity biocatalyst preparation for use in non-aqueous media. It may be added that with the recent work with ionic liquids [24,25] the range of low water containing non-aqueous media continues to expand.

## Experimental

α-chymotrypsin (from Bovine pancreas, Cat. No. C-4129), *N*-acetyl-L-phenylalanine ethyl ester and *N*-benzoyl-L-tyrosine ethyl ester (BTEE) were purchased from Sigma Chemical (St. Louis, MO, USA). Octane, *n*-propanol and acetonitrile (anhydrous grade with water content less than 0.001) were obtained from Sigma Chemical (St. Louis, MO, USA) and J.T. Baker (Phillipsburg, NJ, USA) respectively. Trehalose, sucrose, lactose, PEG-6000, *n*-propanol and tris(hydroxymethyl)aminomethane buffer were obtained from Merck (Mumbai, India). PMDA was obtained from Eastman Kodak Company (Rochester, NY, USA). All solvents were dried over molecular sieves prior to use. All other chemicals employed were of analytical grade.

### Protein concentrations

Protein concentrations were determined according to procedure described by Bradford using bovine serum albumin as the standard [26].

### Precipitation of α-chymotrypsin by n-propanol in the presence of trehalose

α-chymotrypsin was precipitated by *n-*propanol according to previous methods [12,15,27]. The α-chymotrypsin (10 μl of 100 mgml^-1 ^in 10 mM potassium phosphate buffer, pH 7.8) was added to saturated aqueous solutions of trehalose (of different volumes), the total volume was then made up to 100 μl, before being cooled at 4°C. This solution was then added dropwise to dry, chilled (4°C) *n*-propanol (3 ml) with constant shaking (150 rpm). After allowing the suspension to be shaken for 30 min at 4°C, it was then centrifuged (12,000 g) for 5 min at 4°C. The precipitate was then rinsed three times with dry *n*-propanol (chilled), before replacing the *n*-propanol with the desired anhydrous organic solvent. After rinsing the precipitate with organic solvent, the reaction was carried out.

### Transesterification activity

The catalytic activities of α-chymotrypsin precipitates (1 mg of solid enzyme) in organic solvents were determined with reference to the transesterification reaction between *N*-acetyl-L-phenylalanine ethyl ester (10 mM) and *n-*propanol (1 M) in organic solvent (2 ml). The reaction mixture was incubated at 25°C with on an orbital shaker (continuous at 150 rpm). The progress of the reaction was monitored by withdrawing aliquots at different time intervals that were then analysed by HPLC [28].

### HPLC analysis

The samples were analysed by HPLC for the presence of the transesterification product using a ZORBAX SB-C18 column (Agilent Technologies, USA). The eluent consisted of 5 percent glacial acetic acid, 55 percent water and 40 percent acetonitrile, and had a flow rate of 1 ml min^-1^. Detection of the product was carried out with a UV detector at 258 nm.

The amount of trehalose precipitated in the presence of α-chymotrypsin was determined by HPLC using a TSKgel™ column (Supelco, USA). The eluent consisted of 0.1 M of Na_2_SO_4 _in 0.1 M of phosphate buffer (pH 6.7) with a flow rate of 1 ml min^-1^, with detection carried out using an RI detector.

### FT-IR analysis of enzyme preparations

The spectra were recorded on a Nicolet™ FTIR 6700 optical spectrophotometer. Different formulations of α-chymotrypsin were suspended in *n*-propanol. The samples were placed in a demountable cell fitted with zinc selenide windows and 500 μm thick Teflon spacers. All spectra were corrected for solvent background. The data collection was carried out using OMNIC 2.1 software. A total of 250 scans were signal averaged at a resolution of 2 cm^-1 ^[29-31].

## Authors' contributions

KS carried out all the experimental work. MNG was involved in all discussions, interpretation of results and drafting the manuscript.
